# Defensive Effect of Lansoprazole in Dementia of AD Type in Mice Exposed to Streptozotocin and Cholesterol Enriched Diet

**DOI:** 10.1371/journal.pone.0070487

**Published:** 2013-07-31

**Authors:** Rupinder K. Sodhi, Nirmal Singh

**Affiliations:** Pharmacology Division, Department of Pharmaceutical Sciences and Drug Research, Faculty of Medicine, Punjabi University, Punjab, India; Clermont Université, France

## Abstract

The present study investigates the potential of lansoprazole (a proton pump inhibitor and agonist of liver x receptors) in experimental dementia of AD type. Streptozotocin [STZ, 3 mg/kg, injected intracerebroventricular (i.c.v), and high fat diet (HFD, administered for 90 days)] were used to induce dementia in separate groups of Swiss mice. Morris water maze (MWM) test was performed to assess learning and memory of the animals. A battery of biochemical and histopathological studies were also performed. Extent of oxidative stress was measured by estimating the levels of brain reduced glutathione (GSH) and thiobarbituric acid reactive species (TBARS). Brain acetylcholinestrase (AChE) activity and serum cholesterol levels were also estimated. The brain level of myeloperoxidase (MPO) was measured as a marker of inflammation. STZ and HFD produced a marked decline in MWM performance of the animals, reflecting impairment of learning and memory. STZ/HFD treated mice exhibited a marked accentuation of AChE activity, TBARS and MPO levels along with a fall in GSH levels. Further, the stained micrographs of STZ/HFD treated mice indicated pathological changes, severe neutrophilic infiltration and amyloid deposition. Lansoprazole treatment significantly attenuated STZ and HFD -induced memory deficits, biochemical and histopathological alterations. It also prevented HFD-induced rise in the cholesterol level. Therefore, the findings demonstrate potential of lansoprazole in memory dysfunctions which may probably be attributed to its anti-cholinesterase, anti-oxidative and anti-inflammatory effects. Moreover, both cholesterol-dependent as well as cholesterol-independent effects of lansoprazole appear to play a role. In addition study indicates the role of liver x receptors in dementia.

## Introduction

Alzheimer’s disease (AD) is an age-dependent neurodegenerative disease typified by progressive neuronal loss and cognitive impairment. The typical dementia of AD type is characterized by prominent episodic memory impairment, with secondary deficits in word-finding skills, spatial cognition, executive functions and neuropsychiatric changes [1]. It has been demonstrated that 60% of the demented patients manifest typical pathological findings- extraneuronal deposits of β amyloid (Aβ) fibrils (fAβ) and intraneuronal tangles of hyperphosphorylated tau, while a further 15% have these findings accompanied by brain damage of vascular origin [1], [2]. AD brain also exhibits prominent activation of innate immune responses contributing to neuronal loss [2]. Increasing evidences specifically prove that high cholesterol diets increase the risk of sporadic AD [3], [4]. The role of cholesterol metabolism, apolipoprotein E (apoE) and ATP-binding cassette protein A1 (ABCA-1) in AD pathogenesis is undisputed [4], [5]. Since AD pathology is complex and multifactorial, hence it is recommended that managing the disease with a single drug that can modulate the disease pathology from multiple perspectives may prove more effectual. The most effective medications for AD approved by Food and Drug Administration (FDA) are the acetylcholinesterase (AChE) inhibitors, donepezil, galantamine and rivastigmine [3]. Memantine is an N-methyl-d-aspartate (NMDA) receptor antagonist also approved for use in AD and was the first drug approved for treatment of moderate to severe AD [3], [6]. The principal classes of drugs which have reached phase III clinical studies include Rember (tau aggregation inhibitor), Dimebon (mitochondrial stabilizer), bapineuzumab and IgIV (anti-Aβ antibodies), alphatocopherol (antioxidant), docosahexanoic acid (modulates presenelin), Resveratrol (neuroprotective) and Solanezumab (removes Aβ).The liver x receptors α and β (LXRα/NR1H3 and LXRβ/NR1H2) are oxysterol-activated nuclear receptors which play a pivotal role in the control of cellular and whole body cholesterol homeostasis [7]. Studies prove LXR agonists as important therapeutic targets for atherosclerosis through their cholesterol lowering actions [8]. Since there is an enormous correlation between cholesterol content and AD pathology, interest is evoked to unearth the role of LXR in AD pathogenesis. LXR isoforms are expressed in various regions of brain at a significant level, the LXRβ level is 2–5 folds higher than the LXRα [9]. Reports have exhibited the presence of these receptors in cultured neurons, glia, and astrocytes [10]. Essential role of LXRs has been documented in brain structure and function, since aging LXRα/β knockout mice have been described to develop cellular lipid inclusions, abnormalities of choroid plexus closure of lateral ventricles and abnormalities of vasculature [11]. It has been reported that LXRs upregulate the gene expression of ATP-binding cassette transporter A1 (ABCA1), ATP-binding cassette transporter G1 (ABCG1), and human apolipoprotein E gene (APOE) in astrocytes, suggesting a crucial role of LXRs in regulation of brain lipid homeostasis [9]. Reports also exhibit that LXR agonists markedly enhance the clearance of Aβ by promoting the lipidation of apoE in concert with proteinases such as insulin degrading enzyme (IDE) and neprilysin (NEP) [12]. Furthermore studies support that LXRs are potent inhibitors of gene expression of cox2, mcp1 and iNos in glial cell hence reducing the inflammatory responses of these cells towards amyloid β [13]. Lansoprazole is a gastric proton pump inhibitor utilized clinically for the management of acid reflux disease. A recent report based on reporter transactivation assay and secondary invitro assays using established cell lines demonstrate that lansoprazole directly activates the liver x receptors [14]. It has also been documented that lansoprazole increases the ABCA-1 and apoE protein level in mouse derived primary astrocytes [14]. Selective interactions of lansoprazole have also been observed with neurofibrillary tangles composed of tau protein [15].Based on these reports, the present study has been undertaken to explore the potential of lansoprazole in dementia of AD type induced by streptozotocin (STZ) and High fat diet (HFD) in mice.

## Materials and Methods

### Experimental Animals

Swiss albino mice (20–25 g) of either sex (procured from Hisar agriculture university, Hisar, India) were employed in the present study and were housed in the departmental animal house with free access to water and standard laboratory pellet chow diet (Kisan Feeds Ltd. Mumbai, India). They were exposed to 12hr light and 12 hr dark cycle. The animals were acclimatized to the laboratory conditions before experiments. The experiments were performed between 9.30–17.30 h in semi sound proof laboratory conditions. The experimental protocol was duly approved by the institutional animal ethical committee and care of the animals was carried out as per the guidelines of Committee for the Purpose of Control and Supervision of Experiments on Animals (CPCSEA), Ministry of Environment and Forests, Government of India (Reg. No. 107/1999/CPCSEA).

### Drugs and Reagents

All drugs were freshly prepared before use. Lansoprazole (Ranbaxy laboratories Ltd., Toansa, India) was dissolved in dimethyl sulfoxide (1% v/v DMSO). Streptozotocin and 1,1,3,3 tetra-methoxy propane were purchased from Sigma Aldrich, USA. 5,5′-dithiobis (2-nitro benzoic acid) DTNB, Bovine serum albumin (BSA), and reduced Glutathione (GSH) standard were purchased from Sisco Research Laboratories P vt Ltd., Mumbai, India. Thiobarbituric acid was purchased from Loba Chemie, Mumbai, India. Streptozotocin was dissolved in freshly prepared artificial cerebrospinal fluid (ACSF) (147 mM NaCl; 2.9 mM KCl; 1.6 mM MgCl_2_; 1.7 mMCaCl_2_; and 2.2 mM dextrose). Standard Cholesterol estimation kit (Monozyme India Limited, Secunderabad) was used to estimate total serum cholesterol level.

### Laboratory Models

#### Intra cerebroventricular (i.c.v) Streptozotocin (STZ) induced dementia

Mice were anesthetized with anesthetic ether [16]. A polypropylene tube was placed round a hypodermic needle of 0.4 mm external diameter exposing about 3 mm at the tip, and was attached to a 10 µl Hamilton microlitre syringe (Top Syringe, Mumbai, India), which was inserted perpendicularly through the skull (not more than 3 mm) into the brain of mouse. The injection site was 1 mm to right or left midpoint on the line drawn through to the anterior base of the ears. Injections were performed into right and left ventricle on alternate days. Two doses of STZ (3 mg/kg, *i.c.v,* 10 µl each) were administered bilaterally on day 1 and 3. The STZ concentration was adjusted to deliver 10 µl per injection. The injection was made in two locations due to the difficulty of administering 10 µl to a single site. To ascertain that the drug was administered exactly into the cerebral ventricles, some mice (20%) were injected with 5 µl of diluted potent blue dye and their brains were examined macroscopically after sectioning. STZ was dissolved in artificial CSF (25 mg ml^−1^) solution which was made freshly just before the injection. Control mice were administered ACSF *via i.c.v* injection in similar manner.

**Figure 1:**
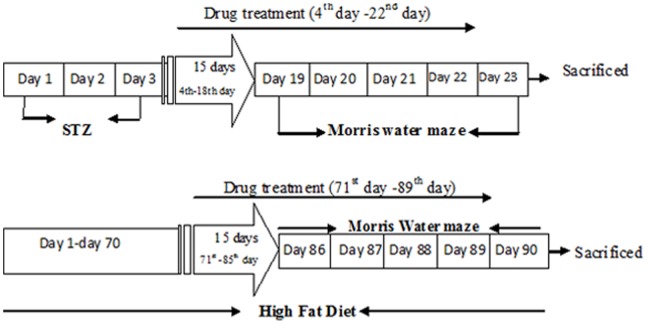
Experimental Protocol.

#### High Fat Diet (HFD) induced experimental dementia

Animals were subject to a cholesterol-rich diet for 90 days and allowed free access to the high fat diet (HFD) 24 hours/day for 90 days to induce memory impairment [17]. High fat diet was prepared by properly mixing the ingredients mentioned in Table 1.

**Table 1:** Composition of High Fat Diet (HFD).

Feed contents			Mass (g)
Powdered pellets diet			365
Lard			310
Casein			250
Cholestrol			10
Sodium cholate			5
DL-methionine			3
Yee-sac powder			1
*Vitamins and Minerals			55
Sodium chloride			1
Total			1000

* Composition of vitamins and mineral mixture per kg of HFD : vitamin A (120000 I.U), vitamin D3 (24000 I.U), vitamin B2 (48 mg), vitamin E (18 units), vitamin K (24 units), calcium pantothenate (60 mg), nicotinamide (240 mg), vitamin B12 (144 mg), calcium (18 g), magnesium (660 mg), iodine (24 mg), iron (180 mg), zinc (360 mg), copper (48 mg), cobalt (108 mg).

#### Morris water maze (MWM) Test

Morris water maze test was employed to assess learning and memory of the animals [18]. MWM is a swimming based model where the animal learns to escape on to a hidden platform. It consisted of large circular pool (150 cm in diameter, 45 cm in height, filled to a depth of 30 cm with water at 28±1°C). The water was made opaque with white colored non-toxic dye. The tank was divided into four equal quadrants with help of two threads, fixed at right angle to each other on the rim of the pool. A submerged platform (10 cm^2^), painted in white was placed inside the target quadrants of this pool, 1 cm below surface of water. The position of platform was kept unaltered throughout the training session. Each animal was subjected to four consecutive training trials on each day with inter-trial gap of 5 min. The mouse was gently placed in the water between quadrants, facing the wall of pool with drop location changing for each trial, and allowed 120 s to locate submerged platform. Then, it was allowed to stay on the platform for 20 s. If it failed to find the platform within 120 s, it was guided gently onto platform and allowed to remain there for 20 s. Day 4 escape latency time (ELT) to locate the hidden platform in water maze was noted as index of acquisition or learning. Animal was subjected to training trials for four consecutive days, the starting position was changed with each exposure as shown in Table 2, and target quadrant (Q 4) remained constant throughout the training period.

**Table 2:** Sequence of training trials on Morris water-maze.

Day 1	Q1	Q2	Q3	Q4
Day 2	Q2	Q3	Q4	Q1
Day 3	Q3	Q4	Q1	Q2
Day 4	Q4	Q1	Q2	Q3

On fifth day, platform was removed and each mouse was allowed to explore the pool for 120 s. Mean time spent in all four quadrants was noted. The mean time spent by the animal in target quadrant searching for the hidden platform was noted as index of retrieval or memory. The experimenter always stood at the same position. Care was taken that relative location of water maze with respect to other objects in the laboratory serving, as prominent visual clues were not disturbed during the total duration of study.

### Biochemical Parameters

Animals were sacrificed by cervical dislocation, brains were removed and homogenized in phosphate buffer (pH = 7.4). The homogenates were than centrifuged (Remi cooling centrifuge; C-24BL) at 3000 rpm for 15 min. The supernatant of homogenates were used for biochemical estimations as per the methods described below. Blood sample was collected by retro-orbital puncture just before sacrificing the animal. The blood was then kept at room temperature for 30 min after which it was centrifuged at 4000 rpm for 15 min to separate serum. Serum was used to estimate the level of serum total cholesterol. While additional brain samples were preserved in 4% neutral formalin for histopathological examination.

#### Estimation of brain acetyl cholinesterase (AChE) activity

The whole brain AChE activity was measured by the method of Ellman et al. (1961) with slight modifications [19]. Change in absorbance per min of the sample was read spectrophotometrically (DU 640B spectrophotometer, Beckman Coulter Inc., CA, USA) at 420 nm.

#### Estimation of brain thiobarbituric acid reactive species (TBARS) level

The whole brain TBARS level was measured by the method of Ohokawa et al. (1979) with slight modifications [20]. The absorbance of developed colour in organic layer was measured spectrophotometrically at 532 nm (DU 640B spectrophotometer, Beckman Coulter Inc., CA, USA). The absorbance from a standard curve generated using 1,1,3,3, tetra-methoxy propane as standard (range = 1 nmol–10 nmol) was extrapolated.

#### Estimation of brain reduced glutathione (GSH) level

The whole brain GSH level was measured by the method of Beutler et al. (1963) with slight modifications [21]. The absorbance was measured spectrophotometrically at 412 nm (DU 640B spectrophotometer, Beckman Coulter Inc., CA, USA). Different concentration of GSH standard was also processed similarly to prepare a standard curve (5–50 µg) simultaneously. Results were expressed as nmole of GSH/mg of protein.

#### Estimation of brain myeloperoxidase (MPO) level

Measurement of myeloperoxidase (MPO) activity was carried out as marker of brain neutrophil infiltration and hence inflammation Green et al., (2004) [22]. Brain myeloperoxidase (MPO) was assayed with the method given by Barone et al. (1991) with slight modifications [23]. MPO activity was studied spectrophotometerically at 460 nm (DU 640B spectrophotometer, Beckman Coulter Inc., CA, USA). MPO activity was expressed as U/G and was calculated according to the given formula

MPO activity (U/G) = X/weight of piece of tissue

Where X = 10×(change in absorbance/min)/volume of supernatant taken in final reaction.

#### Estimation of brain total protein

For the estimation of total protein in brain, method of Lowry et al. (1951) with slight modifications was used [24]. Then absorbance was determined spectrophotometrically at 750 nm (DU 640B spectrophotometer, Beckman Coulter Inc., CA, USA) against suitably prepared blank. A standard curved using 25–200 mg of BSA was plotted. The amount of total protein was expressed in mg.

#### Estimation of serum cholesterol

The total serum cholesterol level was measured by the Allain’s method of CHOD/POD- Phosphotungstate with slight modifications using commercially available conventional diagnostic kit [25].

### Histopathological Examination

#### HE staining

At the end of the experiment brain tissue was fixed in 4% formalin to prevent autolysis and putrefaction. Tissue processing was done according to standard procedures of fixation, dehydration, impregnation, embedding, sectioning and staining with haematoxylin and eosin as described by method of Banchroft [26]. The micrographs of the relevant stained sections (5-µm thickness) were subsequently taken and observed with the aid of a light microscope (at magnification x40). Furthermore, counting of neutrophils per field was performed using a stage micrometer. Arithmetic mean of neutrophils count in 10 fields (Magnified 400 x) was recorded [26].

#### Congo red staining

The sections (5-µm thickness) were dipped in congo red solution for 18 minutes, washed under running tap water for 20 mins, and then placed in weak alkali for 10sec. The sections were incubated in haematoxylin for 5 min, and again washed under running water further standard steps of dehydration; clearing and coverslipping were performed as described by Puchtler [27]. Amyloid-beta deposition was observed under a light microscope (at magnification x100) and analysed using NIH image software.

### Experimental Protocol (Figure 1)

Separate groups of mice were employed in the present study and each group comprised of 10 mice. The drug treatment (Lansoprazole) was given to STZ mice for a total 19 days and was initiated after the second dose of Streptozotocin on the fourth day and continued for 15 days, following which the animals were subjected to the Morris water maze (MWM) test (Day 19–23) for further 5 days. However in the HFD group, the animals were fed with cholesterol rich diet for 90 days. The drug treatment was initiated on the day 71 and continued for 15 days, after which the animals were subjected to the MWM test (Day 86–90) for further days 5 days. In both the models the drug treatment was continued during day 1 to day 4 of acquisition trial on MWM; however vehicle alone was administered on 5^th^ day of the retrieval trial. After the completion of MWM test, the animals were sacrificed; whole brain was removed and used for biochemical and histopathological examination. Blood samples were collected just before sacrificing the animals.

#### Group I: Control group

Mice were administered distilled water (10 ml/kg, *p.o.*) 30 mins before acquisition trial conducted from day 1 to day 4, and 30 min before retrieval trail conducted on day 5.

#### Group II: Artificial cerebrospinal fluid (ACSF) control group

Mice were injected ACSF (25 mg/kg, 10 µL, i.c.v) in two dosage schedules i.e. on the 1^st^ and 3^rd^ day followed by exposure to MWM test after 15 days.

#### Group III: DMSO control group

Mice were administered DMSO (10 ml/kg, *p.o.*) daily for 15 days and then subjected to MWM test. The vehicle was also administered 30 min before acquisition trial conducted from day 1 to day 4 and before retrieval trial conducted on day 5.

#### Group IV: Streptozotocin (STZ) treatment group

Mice were injected Streptozotocin (3 mg/kg, 10 µL, i.c.v) in two dosage schedules i.e. on the first and third day followed by exposure to MWM test after 15 days.

#### Group V: High fat diet (HFD) treatment group

The mice were fed with high fat diet for 90 days and then subjected to Morris water maze test.

#### Group VI: Lansoprazole group

Mice were administered Lansoprazole *per se* (40 mg/kg, *p.o.*) daily for 15 days and again for four consecutive days (day1 to day 4, 30min before) during acquisition trials. On day 5 the animals were administered the vehicle 30 min before the retrieval trial.

#### Group VII and VIII: STZ+Lansoprazole (low and high dose) group

STZ (i.c.v) treated mice were administered lansoprazole (20 mg/kg, 40 mg/kg, *p.o.*) starting after second dose of STZ and then subjected to MWM test. Lansoprazole treatment was also continued (30 min before) during the acquisition trial conducted from day 1 to day 4. The animals were administered the vehicle DMSO (10 ml/kg, *p.o.*) 30 min before the retrieval trial conducted on day 5.

#### Group IX and X: HFD+ Lansoprazole (low and high dose) group

The high fat diet mice were treated with lansoprazole (20 mg/kg, 40 mg/kg, *p.o.*) for 15 days (from day 71– day 85). Lansoprazole treatment was also continued (30 min before) during the acquisition trial conducted from day 1 to day 4 (starting from day 86). The animals were administered the vehicle DMSO (10 ml/kg, *p.o.*) 30 min before the retrieval trial conducted on day 5.

### Statistical Analysis

The results were expressed as mean ± SEM (standard error mean). The data obtained from various groups were statistically analyzed using one-way ANOVA followed by Tukey’s multiple range test. P value <0.05 was considered to be significant.

## Results

### Effect on ELT and Time Spent in Target Quadrant (TSTQ) Using Morris Water Maze

**Figure 2:**
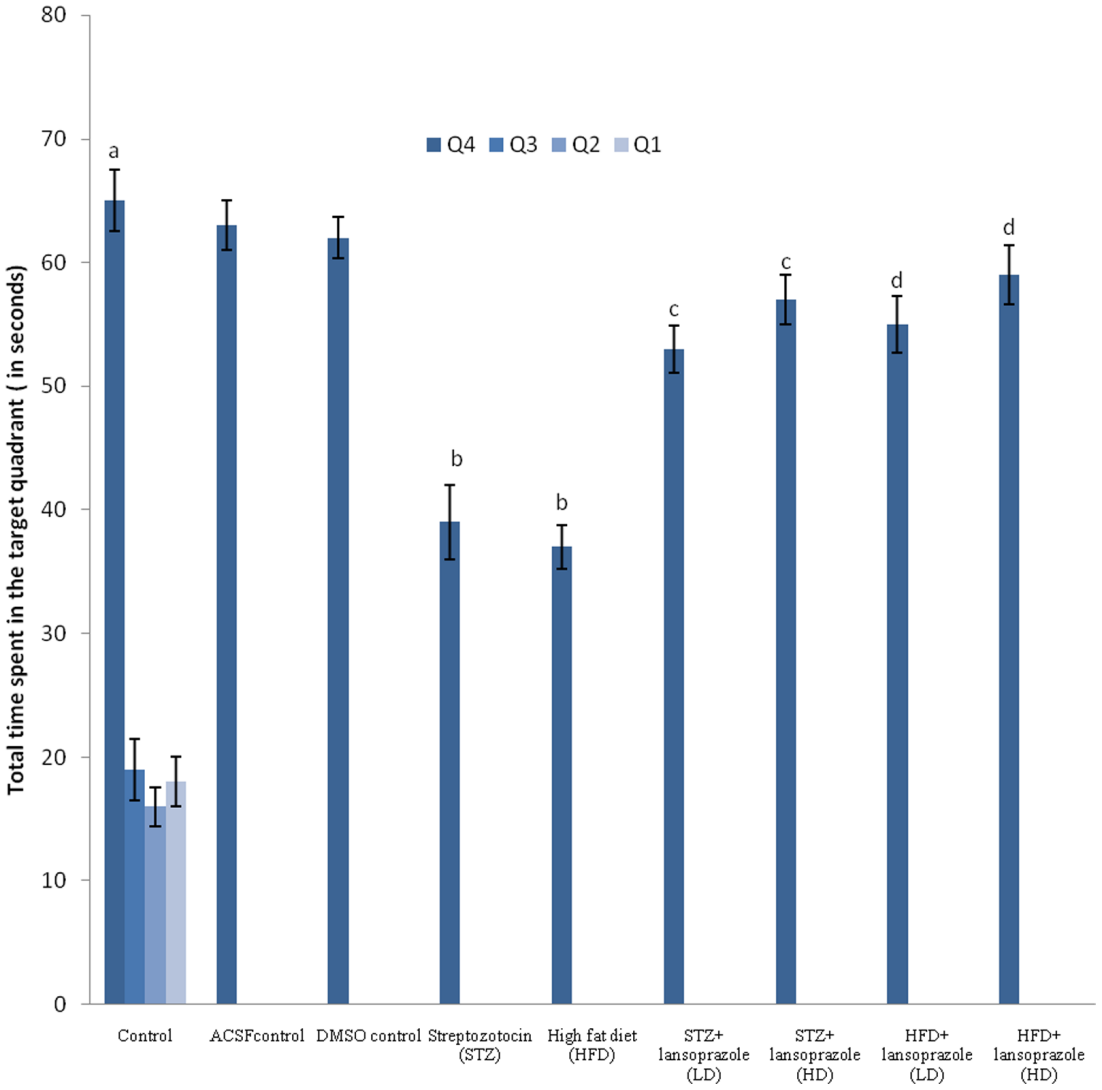
Effect of pharmacological interventions on Total time spent in the target quadrant in seconds (TSTQ) using Morris water maze test. Each group (n = 10) represents mean±S.E.M. a = p<0.05 Vs time in other quadrants in control group, b = p<0.05 Vs time spent in target quadrant in control group, c = p<0.05 Vs time spent in target quadrant in STZ control group, d = p<0.05 Vs time spent in target quadrant in HFD control group. ANOVA followed by Tukey’s multiple range test. LD = low dose, HD = high dose.

Control untreated mice exhibited a significant fall in day 4 ELT as compared to its value on day 1 (Table 3). Further significantly more time was spent in the target quadrant (Q4) in search of the missing platform as compared to the total time spent in the other quadrants (Q1, Q2 and Q3) during the retrieval trial on day 5 (Figure2). Mice treated with vehicle DMSO, did not exhibit any significant difference on day 4 ELT (Table 3) and day 5 TSTQ as compared to the control group (Figure 2). Administration of STZ (3 mg/kg, 10 µL, I.C.V) and HFD for 90 days, significantly prevented the decrease in day4 ELT as compared to the control group (Table 3) and markedly diminished TSTQ (Q4) observed in the retrieval trial on day5 (Figure 2). Further, Lansoprazole (40 mg/kg *p.o.*) *per se* did not show any significant change in day 4 ELT and day 5 TSTQ in comparison to the control group (Table 3 and Figure 2). Treatment with Lansoprazole (20 mg/kg and 40/kg, *p.o.*) significantly attenuated the day 4 rise in ELT (Table 3) and decrease in day 5 TSTQ in the STZ as well as HFD treated mice in a dose dependent manner (Figure2).

**Table 3:** Effect of various therapeutic interventions on Escape latency time Day 1 and Day 4 on Morris water maze (in seconds).

Group	Treatment	day1 ELT (seconds)	day 4 ELT (seconds)
I	Control (untreated)	83±2.3	38±2.8^a^
II	ACSF control	85±3.2	35±3.5
III	DMSO control	82±2.5	35±2.4
IV	Streptozotocin (STZ)	107±2.3	84±3.2^b^
V	High Fat diet (HFD)	108±2.4	85±2.7^b^
VI	Lansoprazole per se	86±4.1	34±2.9
VII	STZ+ Lansoprazole (Low dose)	85±2.0	40±2.4^c^
VIII	STZ+ Lansoprazole (High dose)	86±3.2	34±2.2^c^
IX	HFD+ Lansoprazole (Low dose)	85±2.8	37±3.2^d^
X	HFD+ Lansoprazole (High dose)	83±2.3	33±2.6^d^

Each group (n = 10) represents mean±S.E.M. a = p<0.05 as compared to the day 1 ELT in control, b = p<0.05 as compared to the day 4 ELT, in control, c = p<0.05 as compared to the day 4 ELT, in STZ control, d = p<0.05 as compared to the day 4 ELT, in HFD control ANOVA followed by Tukey’s multiple range test.

### Effect on Brain AChE Activity

i.c.v STZ and HFD administration significantly, increased the brain AChE activity when compared to the control group (Table 4). Lansoprazole (40 mg/kg *p.o.*) *per se* did not produce any significant effect on brain AChE level as compared with the control group (Table 4). However Lansoprazole (20 mg/kg and 40/kg, *p.o.*) significantly reduced the STZ and HFD- induced increase in AChE activity (Table 4).

**Table 4:** Effect of therapeutic interventions on various biochemical parameters.

Group	Treatment	Brain AchE activity (µMof Ach hydrolysed/min/mgof protein)	Brain TBARS(nM/mg protein)	Brain GSH(µM/mg protein)	Brain MPO (U/mgof protein)	Serumcholesterol(mg/dl)
I	Control	1.5±0.5	5.2±0.88	26.8±2.9	0.05±0.01	120±2.6
II	ACSF control	1.45±0.043	4.8±0.9	21.6±2.7	0.04±0.015	123±3.4
III	DMSO control	1.46±0.07	4.9±0.98	22.2±1.8	0.03±0.01	118±2.0
IV	Streptozotocin (STZ)	3.2±0.08^a^	18.8±1.3^a^	7.8±1.5^a^	0.32±0.8^a^	121±1.3
V	High Fat diet (HFD)	2.9±0.05^a^	19.2±0.8^a^	8.3±2.3^a^	0.35±0.015^a^	224±3.2^a^
VI	Lansoprazole per se	1.5±0.07	5.7±1.4	25.2±1.8	0.05±0.02	119±1.8
VII	STZ+ Lansoprazole(low dose)	1.46±0.06^b^	9.3±1.1^b^	12.5±1.7^b^	0.13±0.017^b^	119±1.9
VIII	STZ+ Lansoprazole(high dose)	1.42±0.08^b^	8.6±0.97^b^	22.4±1.4^b^	0.09±0.02^b^	121±2.2
IX	HFD+ Lansoprazole(low dose)	1.43±0.06^c^	10.9±1.3^c^	14.3±1.6^c^	0.08±0.01^c^	137±1.8^c^
X	HFD+ Lansoprazole(high dose)	1.40±0.05^c^	8.2±2.1^c^	18±1.8^c^	0.06±0.02^c^	125±2.1^c^

Each group (n = 10) represents mean ±S.E.M.

a = p<0.05 Vs control,

b = p<0.05 Vs STZ control,

c = p<0.05 Vs HFD control. ANOVA followed by Tukey’s multiple range test.

### Effect on Brain Oxidative Stress Levels

i.c.v STZ and HFD administration markedly, accentuated the brain TBARS level (Table 4) and reduced the brain GSH levels (Table 4) in comparison to the control group animals exhibiting enhanced oxidative stress. Lansoprazole (40 mg/kg *p.o.*) *per se* did not produce any significant effect on brain oxidative stress as compared with the control group (Table 4). However significant improvement in oxidative stress level in terms of reduced TBARS level and enhanced GSH level was observed with Lansoprazole (20 mg/kg and 40/kg, *p.o.*) in the STZ and HFD mice in a dose dependent manner (Table 4).

### Effect on Brain MPO Levels

i.c.v STZ and HFD significantly, increased the brain MPO levels when compared to the control group. Lansoprazole (40 mg/kg *p.o.*) *per se* had no significant effect the brain MPO level (Table 4). Lansoprazole (20 mg/kg and 40/kg, *p.o.*) significantly reduced the STZ and HFD- induced rise in brain MPO level (Table 4).

### Effect on Serum Cholesterol Level

i.c.v STZ and Lansoprazole (40 mg/kg) *per se* did not show any significant change in the serum cholesterol level. However the HFD mice exhibited a marked rise in the serum cholesterol level when compared to the control group. Further no change was observed in the cholesterol level of lansoprazole -treated STZ mice (Table 4). However a significant decline was observed in the cholesterol level of lansoprazole- treated HFD mice.

### Effect on Body Weight

Mice treated with HFD for 90 days (Table 5) exhibited a significant increase in the body weight, when compared to the body weight of the control animals fed on normal diet. However, i.c.v STZ administration did not show significant variation in the body weight in comparison to the control mice. Further HFD mice treated with lansoprazole (20 mg/kg and 40/kg, *p.o.*) showed a marked decrease in the body weight in comparison to the control animals (Table 5).

**Table 5:** Effect of therapeutic interventions on body weight of mice.

Group	Treatment	Body weight (g) Basal value	Body weight (g) Final value
I	Control	24.0±0.25	24.68±0.29
II	ACSF control	24.13±0.37	24.22±0.26
III	DMSO control	24.20±0.28	24.36±0.22
IV	Streptozotocin (STZ)	24.33±0.26	24.15±0.31
V	High Fat diet (HFD)	23.36±0.21	31.34±0.26^a^
VI	Lansoprazole per se	23.92±0.41	24.10±0.39
VII	STZ+ Lansoprazole (low dose)	24.81±0.33	24.79±0.40
VIII	STZ+ Lansoprazole (high dose)	24.75±0.25	24.95±0.27
IX	HFD+ Lansoprazole (low dose)	24.19±0.21	27.3±0.50^b^
X	HFD+ Lansoprazole (high dose)	24.27±0.26	28.2±0.29^b^

Each group (n = 10) represents mean ± S.E.M.

a = p<0.05 Vs body weight in control,

b = p<0.05 Vs body weight in HFD treated group. ANOVA followed by Tukey’s multiple range test.

### Histopathological Changes in Brain

**Figure 3:**
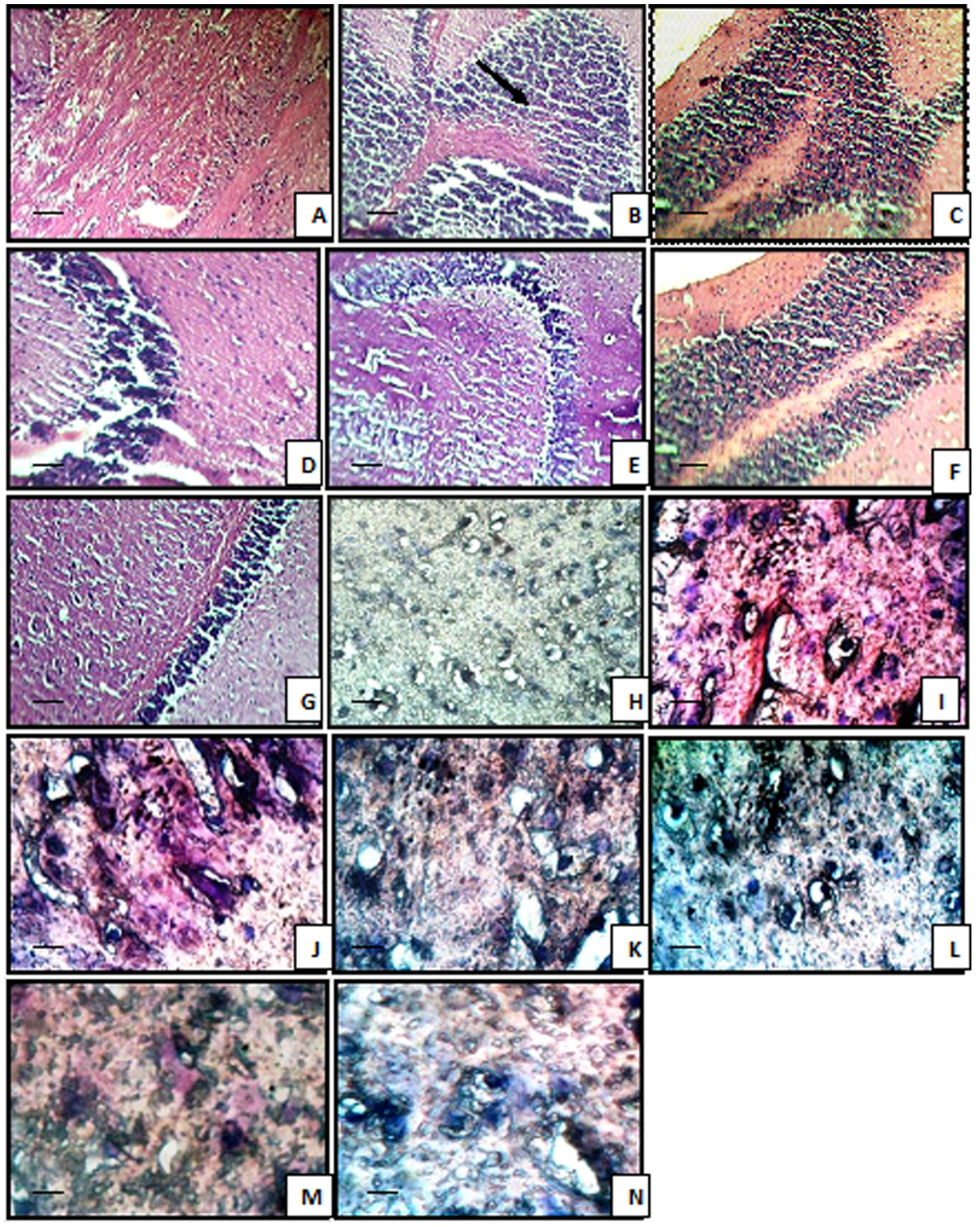
Microscopic study of mice brain. Histological sections of brain were stained with hematoxylin and eosin (H&E) and Congo red. Control (A) showing normal histological features. STZ-treated and HFD mice (B and C) showing focal as well as diffuse severe neutrophilic infiltration. Lansoprazole (20 mg/kg, 40 mg/kg) treated STZ mice (D and E) and Lansoprazole (20 mg/kg, 40 mg/kg) treated HFD mice (F and G) featuring mild neutrophilic infiltration and pericellular edema (A–G, HE staining, grossly ×400). Control (H) Congo red deposition was not detected. STZ-treated and HFD mice (I and J) showing orange-red deposits of Congo red. Lansoprazole (20 mg/kg, 40 mg/kg) treated STZ (K and L) and HFD mice (M and N) showing mild Congo red deposits (F–I, Congo red staining, grossly ×1000). Scale bar 35 µm.

**Figure 4:**
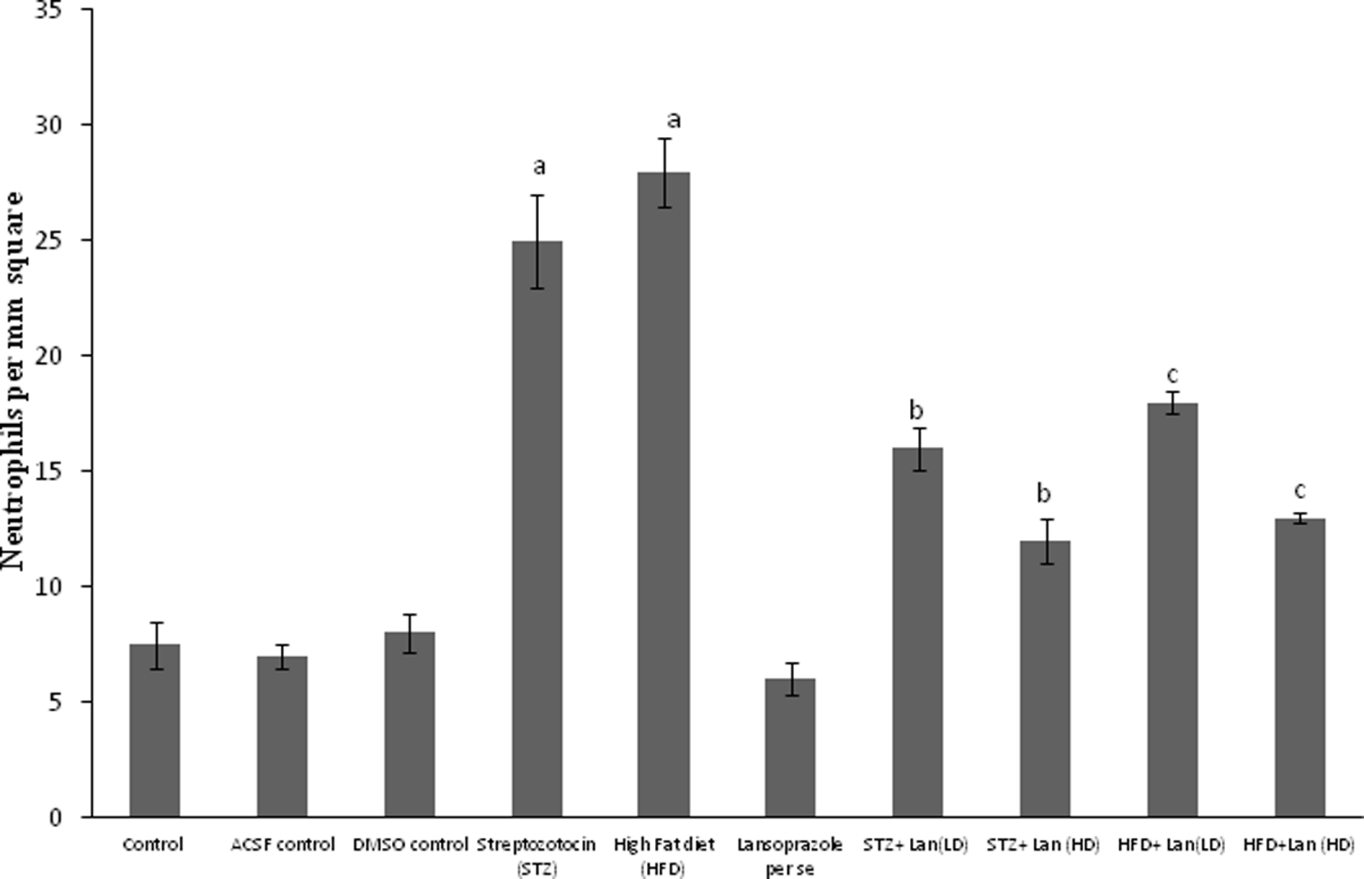
Effect of pharmacological interventions on neutrophil infiltration. Each group (n = 10) represents mean±S.E.M. a = p<0.05 Vs control group, b = p<0.05 Vs STZ control group, c = p<0.05 Vs HFD control group. ANOVA followed by Tukey’s multiple range test. Lan = Lansoprazole, LD = low dose, HD = high dose.

The stained micrographs exhibited that control mice brain coronal sections presented a normal brain histological pattern (Figure 3). On the contrary, STZ and HFD treated mice revealed severe focal as well as diffuse neutrophilic infiltration, accompanied by dilatation of blood vessels and pericellular edema as observed with H&E staining method. Histopathological examination of lansoprazole (20, 40 mg/kg, *p.o.*) treated STZ and HFD mice, showed mild neutrophilic infiltration and mild pericellular edema (Figure 3, 4). Furthermore STZ as well as HFD treated animals exhibited positive congo red staining observed as orange-red colouration signifying β-amyloid deposition. Reduced congo red deposition was observed in lansoprazole-treated, STZ and HFD groups (Figure 3).

## Discussion

**Figure 5:**
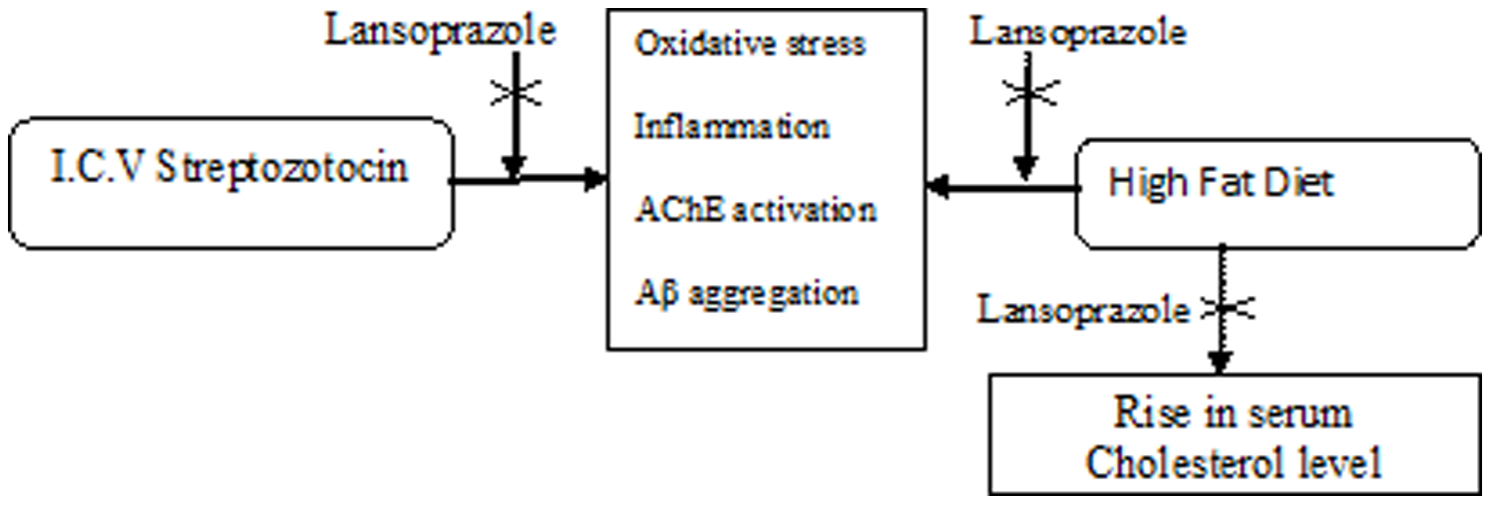
Proposed mechanism for neuroprotective effect of lansoprazole.

Results of the present investigation indicate that intracerebroventricular (i.c.v) administration of streptozotocin (STZ, 3 mg/kg) and High fat diet (HFD) for 90 days has produced cognitive deficits, abnormal biochemical alterations and morphological changes in the mice brain similar to that of dementia of AD type. Further HFD also accentuated the body weight and serum cholesterol levels in a significant manner. Treatment of lansoprazole produced a protected effect on STZ/HFD induced cognitive, biochemical and histopathological changes (Figure 5).

Our previous reports have indicated, STZ (i.c.v) as a well documented animal model of dementia of Alzheimer’s type, typically producing prolonged impairment of brain energy metabolism and oxidative stress [28], [29]. Damage to myelin sheath by oxidative stress and inhibition of Adenosine triphosphate (ATP) and acetyl Co-A synthesis by STZ may lead to cognitive dysfunction [30], [31]. Further it has also been reported that i.c.v STZ causes brain inflammation by up regulation of some genes in the brain viz. brain-derived neurotrophic factor, glial cell derived neurotropic factor and integrin-alpha-M [32]. It is also evidenced that i.c.v STZ leads to the development of oxidative stress in brain [16].

Studies have documented that administration of cholesterol rich high fat diet induces memory deficits in rodents [33], [34]. HFD model has been a well described animal model of dementia typically characterised by increased expression of cytokines, chemokines and increased reactive astrocytosis and microgliosis triggering brain inflammation [35]. Increased brain cholesterol has also been documented to raise the β amyloid peptide, PGE_2_ production, activation of NF-κB in brain eventually culminating in neuronal damage and dementia [36], [37]. Specifically it still remains unclear whether a modulatory effect on brain cholesterol metabolism can alter the development and progression of AD pathology. Clinical studies have suggested that the net brain cholesterol concentration is regulated by the serum cholesterol level and there is a cross-talk between the CNS and peripheral cholesterol pools [38]. Therefore, it is plausible that peripheral cholesterol levels modulate CNS cholesterol levels and vice versa. In line with above studies, STZ and HFD treated mice in our study performed poorly on Morris water maze indicating impairment in their learning abilities and memory capabilities. Further there was a significant rise in brain acetyl cholinesterase (AChE) activity and brain oxidative stress levels (indicated by an increase in TBARS and decrease in GSH levels) which is consistent with earlier reports [39] [40]. A marked rise in the level of brain MPO was also detected in STZ and HFD-treated mice indicative of neuroinflammation. In our study the brains of STZ and HFD-treated animals, studied by optical microscopy, displayed massive neutrophilic infiltration, pericellular edema and congestion of blood vessels, indicative of accelerated pathological and inflammatory events by STZ. Cardinal pathological lesion encountered in Alzheimer’s disease is the presence of neuritic plaques composed of a central core of a 4-kD protein with a beta-pleated sheet configuration called beta-amyloid. The predominant beta-pleated sheet configuration of beta-amyloid confers it the ability to bind the planar dye congo red and produce an orange-red coloration when seen under light microscope and apple-green birefringence under polarized light [41]. In our study optic micrographs of STZ and HFD treated brain sections, illustrate positive congo red staining indicative of amyloid deposition.

In the present study, administration of lansoprazole, significantly defended the memory deficits produced by STZ and HFD (Figure 5). In addition it also restored the AChE activity, attenuated oxidative alterations and brain MPO level in a dose dependent manner. Furthermore, treatment of lansoprazole to STZ and HFD exposed animals, exhibited reduced histopathological alterations, illustrated by mild neutrophilic infiltration, and pericellular edema, that suggests marked protective effect and anti-inflammatory effect of lansoprazole against the toxic effects of STZ and high cholesterol administration. The deposition of congo red was also reduced in lansoprazole -treated STZ and HFD brain sections. These findings indicate that lansoprazole may contribute towards prevention of amyloid deposition in brain.

Reports have documented essential role of LXRs in brain structure and function [11]. Experiments reveal that LXR knockout mice exhibit adult onset motor degeneration which is accompanied by axonal dystrophy, astrogliosis and lipid accumulation [11]. The potential of LXRs as attractive targets in AD arises from their ability to modulate important determinants of AD such as cholesterol concentration, apolipoprotein E, amyloid β and inflammation. Studies utilizing primary neurons, astrocytes and microglia isolated from embryonic rat brain illustrate that LXR activation augments the expression of target genes involved in cholesterol homeostasis such as *abca-1*, *abcg-1* and *apoe* [42]. It has been suggested that LXR activation reduces Aβ levels by inhibiting APP (amyloid precursor protein) processing through its cholesterol lowering action. Reports reveal that ApoE and its lipidation status play a significant role in brain Aβ deposition and clearance. Outcomes of various studies prove that LXR agonists enhance the ABCA-1 and apoE expression hence reducing the Aβ production in neuro2A cells expressing human APPswe [43]. Essential role of LXRs has been studied in APP/PS1 transgenic mouse model, where LXR activation decreased the amyloid burden and improved memory [44], [12] LXR activation has also been shown to suppress amyloid deposition and marked impairment of memory in APP23 mice induced by high fat diet [45] Activation of LXRs has further been demonstrated to produce anti-inflammatory actions [13] Treatment of AD mouse with LXR agonists documented to induce suppression of microglia activation and potent inhibition of cox2, mcp1 as well as iNos in glial cells hence exerting anti-inflammatory actions [46].

A previous report has demonstrated that lansoprazole inhibits both rat as well as human serum cholinesterase in a concentration dependent manner [47]. Results of different studies also document anti-inflammatory and neuroprotective potential of lansoprazole in Interferone- γ and LPS induced neurotoxicity in human microglia and astrocytes [48], [49]. Lansoprazole has also been reported to reduce tissue oxidative stress induced by non steroidal anti-inflammatory drugs [50], [51]. Therefore, with support from literature and data in hand it is suggested that lansoprazole may have a neuroprotective potential in dementia of AD type in terms of cognition, biochemical and histopathological parameters in mice evoked by STZ and HFD.

### Conclusion

It is concluded that defensive effect of lansoprazole in mouse models of dementia of AD type is attributed to its neuroprotective, anti-cholinesterase, anti-oxidative, anti-inflammatory and amyloid lowering potential. Both, cholesterol-dependent as well as cholesterol-independent effects of lansoprazole appear to play a role. In addition, study highlights the potential of liver-X receptor agonists in dementia of AD. Perhaps this is the first report documenting beneficial effect of lansoprazole in dementia of AD type; nevertheless in depth further studies are required to explore full potential and precise mechanism behind its neuroprotective actions.
